# Three-Dimensional Terahertz Coded-Aperture Imaging Based on Single Input Multiple Output Technology

**DOI:** 10.3390/s18010303

**Published:** 2018-01-19

**Authors:** Shuo Chen, Chenggao Luo, Bin Deng, Hongqiang Wang, Yongqiang Cheng, Zhaowen Zhuang

**Affiliations:** School of Electronic Science, National University of Defense Technology, Changsha 410073, China; dengbin_nudt@163.com (B.D.); oliverwhq@tom.com (H.W.); nudtyqcheng@gmail.com (Y.C.); zwzhuang@nudt.edu.cn (Z.Z.)

**Keywords:** coded-aperture imaging, terahertz, three-dimensional (3D), single input multiple output (SIMO)

## Abstract

As a promising radar imaging technique, terahertz coded-aperture imaging (TCAI) can achieve high-resolution, forward-looking, and staring imaging by producing spatiotemporal independent signals with coded apertures. In this paper, we propose a three-dimensional (3D) TCAI architecture based on single input multiple output (SIMO) technology, which can reduce the coding and sampling times sharply. The coded aperture applied in the proposed TCAI architecture loads either purposive or random phase modulation factor. In the transmitting process, the purposive phase modulation factor drives the terahertz beam to scan the divided 3D imaging cells. In the receiving process, the random phase modulation factor is adopted to modulate the terahertz wave to be spatiotemporally independent for high resolution. Considering human-scale targets, images of each 3D imaging cell are reconstructed one by one to decompose the global computational complexity, and then are synthesized together to obtain the complete high-resolution image. As for each imaging cell, the multi-resolution imaging method helps to reduce the computational burden on a large-scale reference-signal matrix. The experimental results demonstrate that the proposed architecture can achieve high-resolution imaging with much less time for 3D targets and has great potential in applications such as security screening, nondestructive detection, medical diagnosis, etc.

## 1. Introduction

Terahertz waves (0.1–10 THz) have many advantages such as stronger penetration capability than light and higher resolution over microwave, allowing visualization of a hidden object at millimeter level. Traditional approaches for THz imaging, such as active electronically scanned antennas (AESAs) [1,2,3] and synthetic aperture radar (SAR) systems [4,5], cannot strike a balance in low-cost hardware and fast sampling time.

Fortunately, the limitations associated with conventional approaches can be avoided by a more abstract perspective of the measurement process. A new cutting-edge imaging method, THz coded-aperture imaging (TCAI) [6,7,8], provides the mathematical foundation for advanced imaging systems that can take advantage of arbitrary measurement modalities. Based on the basic principles of optical coded-aperture imaging [9,10] and radar coincidence imaging (RCI) [11,12,13], this imaging technology adopts an electrical dynamic aperture to either measure or produce spatiotemporal independent signals in the imaging area, and then resolves the target scatters by using the pseudo-randomness of the signals. Moreover, metasurfaces have shown great promise in flexible manipulation on terahertz and millimeter waves, which have been applied to areas of high-resolution hologram [14,15] and some other functional devices [16,17]. Therefore, as an important technical approach, metasurfaces have increasing abilities in designing coded apertures for the TCAI system [18,19].

Because traditional high-resolution TCAI contains only one receiver, it will take a lot of time on coding and sampling. Besides, common TCAI approaches rely too much on the computing power, which limits its wide application in areas such as security screening, nondestructive detection and medical diagnosis [1,20,21,22]. Especially for 3D human-scale targets, the coding and computing burden becomes serious for current TCAI architecture.

In this paper, we propose a new TCAI architecture based on single input multiple output (SIMO) technology and transmission-type coded aperture. Different from other TCAI architecture, the proposed TCAI architecture achieves rapid imaging by using multiple detecting technology. To reduce the computing burden on large-scale reference-signal matrix, we propose an imaging method combining digital beam scanning and multi-resolution imaging together. Moreover, by inserting the random modulation in the receiving process rather than the transmitting one, we will not damage the irradiation-beam pattern, and thus improve the working distance.

This paper is organized as follows. In Section 2, the basic imaging principle of TCAI is introduced at first. In the adoption of SIMO technology, we design a new TCAI architecture, and deduce its mathematical imaging model. In Section 3, a practical scanning method is proposed to scan the whole 3D imaging area step by step. Moreover, to improve the imaging efficiency, a multi-resolution imaging method is advanced. In Section 4, numerical experiments are implemented to demonstrate the scanning and imaging ability of our TCAI architecture for the 3D human target. Moreover, for the approximate imaging results, the single input single output (SISO) TCAI system consumes much more time. Finally, we summarize the main advantages of our architecture and conclude with future directions and outlook in Section 5.

## 2. Principle and Model

### 2.1. Conventional SISO TCAI Architecture

**Figure 1:**
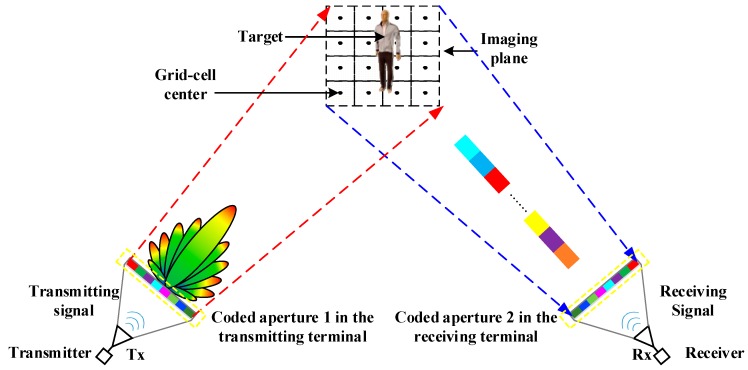
Schematic diagram of the terahertz coded-aperture imaging (TCAI) system.

As shown in the schematic diagram in Figure 1, the common TCAI system is composed mainly of a transmitter, a receiver, coded aperture 1 in the transmitting terminal, and coded aperture 2 in the receiving terminal.

The common TCAI system is mainly based on the SISO structure. The coded aperture, controlled by the computer, can modulate the amplitudes or phases of the input signals. Besides, it can be placed in either one of the transmitting and the receiving terminal, or in both. The imaging plane is divided into *K* grid cells, while the scatters are assumed to be at the center of each grid cell. Herein, the grid cell can be interpreted as resolution volume cell.

Eventually, the imaging target is reconstructed by solving the matrix equation, which is shown as below,

(1) $$\begin{array}{l} \;Sr=S\cdot \beta +w\\ \left[\begin{array}{c} Sr\left(t_{1}\right)\\ Sr\left(t_{2}\right)\\ \ldots \\ Sr\left(t_{N}\right) \end{array}\right]=\left[\begin{array}{cccc} S\left(t_{1},r_{1}\right) & S\left(t_{1},r_{2}\right) & \ldots & S\left(t_{1},r_{K}\right)\\ S\left(t_{2},r_{1}\right) & S\left(t_{2},r_{2}\right) & \ldots & S\left(t_{2},r_{K}\right)\\ \ldots & \ldots & \ldots & \ldots \\ S\left(t_{N},r_{1}\right) & S\left(t_{N},r_{2}\right) & \ldots & S\left(t_{N},r_{K}\right) \end{array}\right]\cdot \left[\begin{array}{c} \beta_{1}\\ \beta_{2}\\ \ldots \\ \beta_{K} \end{array}\right]+\left[\begin{array}{c} w_{1}\\ w_{2}\\ \ldots \\ w_{N} \end{array}\right] \end{array}$$

where $Sr=\left(Sr\left(t_{n}\right)\right),n=1,2,\cdots N$ and $S=\left(S\left(t_{n},r_{k}\right)\right),k=1,2,\cdots K,n=1,2,\cdots ,N$ are the receiving signal vector and reference-signal matrix, respectively [12]. $\beta =\left(\beta_{k}\right),k=1,2,\cdots K$ is the scattering coefficients vector, where K is the number of the grid cells. $w=\left(w_{n}\right),n=1,2,\cdots ,N$ is the additive measurement noise at the receiving terminal. Besides, ${\left\{r_{k}\right\}}_{k=1}^{K}$ are the position vectors of the grid cells on the imaging plane, and ${\left\{t_{n}\right\}}_{n=1}^{N}$ are the time-domain samples. With the knowledge of solving linear equations such as Equation (1), the resolving ability mainly depends on the correlation between different rows and columns of the reference-signal matrix S. An ideal reference-signal matrix is one in which the rows and columns are independent of each other. Our proposed architecture can improve the imaging efficiency without deteriorating its resolving ability. Moreover, it can also reduce the computational complexity by digital beam-scanning technology.

### 2.2. Proposed SIMO TCAI Architecture

**Figure 2:**
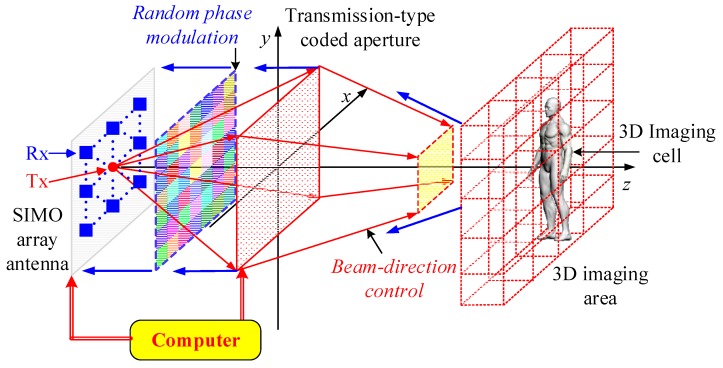
The TCAI architecture for close objects based on transmission coded-aperture.

Inspired by SIMO technology, we design a TCAI system for 3D target based on transmission-type coded aperture, which is shown in Figure 2. The system is mainly composed of a SIMO array antenna, a transmission-type coded aperture and a computer.

On the SIMO array antenna, the red point denotes the single transmitter while the blue squares show the receiving-detector array. Moreover, the transmitting and receiving signals share the same route. As shown in Figure 2, the red and blue flow lines denote the transmitting and receiving processes, respectively. The coded aperture can modulate the phase of the terahertz wave to control its beam direction and spatiotemporal independence in the transmitting and receiving processes, separately. To reduce the computational complexity, we divide the whole 3D imaging area into several 3D imaging cells with the same size, for instance, the yellow cube that is shown in the 3D imaging area.

**Figure 3:**
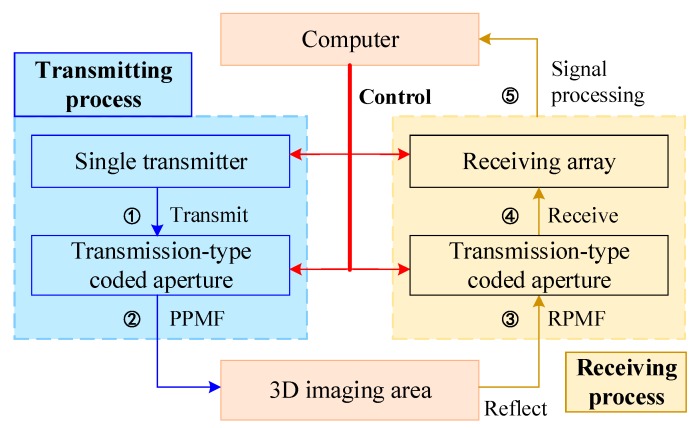
The imaging process of the proposed single input multiple output (SIMO) TCAI architecture.

Figure 3 illustrates the whole imaging process of the proposed TCAI architecture. In the transmitting process, the coded aperture loads purposive phase modulation factor (PPMF) to direct the terahertz beam, which is transmitted from the single transmitter. After reflecting from the 3D imaging area, the signal comes into the receiving process. To modulate the reflected signal randomly for high-resolution performance, the same coded aperture loads the random phase modulation factor (RPMF). Eventually, the receiving array receives the reflected signals and transfers them to the computer for signal processing. Additionally, computer can control the SIMO array antenna and the transmission-type coded aperture simultaneously. Next, according to Figure 3, we deduce the SIMO TCAI imaging model in detail.

**Step 1. Signal Transmitting**

During the transmitting process, the single transmitter transmits a terahertz random frequency-hopping (FH) signal, which is shown as below:

(2) $$S\left(t_{n}\right)=A\exp\left\{j2\pi \left(f_{c}+f_{t_{n}}\right)t_{n}\right\}$$

where $S\left(t_{n}\right)$ is the transmitting signal at time $t_{n}$, $f_{c}$ is the carrier frequency, $f_{t_{n}}$ is the random hopping frequency at time $t_{n}$, and A is the amplitude of the signal.

The transmission-type coded aperture contains array elements of P rows and Q columns. In the transmitting process, we use coded-aperture array element (p,q) to describe the element in the *p*-th row, *q*-th column, while p=1,2,⋯P, and q=1,2,⋯Q. The transmitting signal arriving at the coded-aperture array element (p,q) can be expressed as,

(3) $$S_{p,q}(t_{n})=A\exp\left\{j2\pi \left(f_{c}+f_{t_{n}}\right)\left(t_{n}-t_{Tx,p,q}\right)\right\}$$

where $t_{Tx,p,q}$ is the time delay result from the distance between the coded-aperture array element (p,q) and the transmitter.

**Step 2. PPMF for Digital Beam Scanning**

Controlled by the computer, the PPMF acts as a digital lens to lead the terahertz wave to cover the aimed 3D imaging cell, and it is described as,

(4) $$\varphi_{p,q}^{\mathrm{purpose}}=e^{-i\frac{k}{2f_{\mathrm{focus}}}\left[{\left(x_{p,q}-x_{0}\right)}^{2}+{\left(y_{p,q}-y_{0}\right)}^{2}\right]}$$

where $f_{\mathrm{focus}}$ is the focal length of the designed digital lens. The word “purpose” means controlling the beam direction on purpose with designed phase modulation. $k=2\pi f/c_{l}$ is the wave number of the terahertz wave, f is the transmitting terahertz frequency, $c_{l}$ is velocity of light. $\left(x_{p,q},y_{p,q}\right)$ is the coordinate of the coded-aperture array element (p,q) on the *xoy* plane. $\left(x_{0},y_{0}\right)$ is the coordinate of the phase center for the digital lens.

Suppose the imaging area consists of K grid cells, after transmitting all coded-aperture elements, the detecting signal of the *k*-th grid cell at time $t_{n}$ is expressed as,

(5) $$S_{I}(t_{n},r_{k})=\sum_{p=1}^{P}\sum_{q=1}^{Q}S_{p,q}(t_{n}-t_{p,q,k})\cdot \exp\left(j\cdot \varphi_{p,q}^{\mathrm{purpose}}\right)$$

where $t_{p,q,k}$ is the time delay result from the distance between the coded-aperture array element (p,q) and the *k*-th grid cell.

**Step 3. RPMF for High Resolution Imaging**

Then, the terahertz wave is reflected from the 3D target, and it comes to the receiving process. According to the basic principle of TCAI, the imaging resolution is mainly influenced by the random modulation on terahertz wave. Herein, to achieve high-resolution imaging, the computer controls the coded-aperture array element $\left(p^{\prime },q^{\prime }\right)$ to loads RPMF, which can be expressed as,

(6) $$\varphi_{p^{\prime },q^{\prime }}^{\mathrm{random}}=\mathrm{random}\left(p_{l},p_{h},p^{\prime },q^{\prime }\right)$$

where $p_{l}$ and $p_{h}$ describe the lower and high limits of the phase shift interval, respectively. Herein, each coding element can modulate the phase between $\left[p_{l},p_{h}\right]$ continuously. Different from the transmitting process, we use coded-aperture array element $\left(p^{\prime },q^{\prime }\right)$ to replace (p,q) in the receiving process.

After loading with RPMF, the reflected signal passing through the coded-aperture array element $\left(p^{\prime },q^{\prime }\right)$ reads,

(7) $$Sr_{p^{\prime },q^{\prime }}(t_{n})=\left[\sum_{k=1}^{K}\beta_{k}\cdot S_{I}(t_{n}-t_{k,p^{\prime },q^{\prime }},r_{k})\right]\cdot \exp\left(j\cdot \varphi_{p^{\prime },q^{\prime }}^{\mathrm{random}}\right)$$

where $t_{k,p^{\prime },q^{\prime }}$ is the time delay result from the distance between the *k*-th grid cell and the coded-aperture array element $\left(p^{\prime },q^{\prime }\right)$.

**Step 4. Signal Receiving**

As the SIMO array antenna consists of U rows, V columns of array elements for signal receiving, the antenna-array element in the *u*-th row, *v*-th column can be indexed as antenna-array element (u,v). Thus, the reflected signal arriving at the antenna-array element (u,v) is written as,

(8) $$Sr^{u,v}(t_{n})=\sum_{p^{\prime }=1}^{P}\sum_{q^{\prime }=1}^{Q}Sr_{p^{\prime },q^{\prime }}(t_{n}-t_{p^{\prime },q^{\prime },u,v})=\sum_{k=1}^{K}\beta_{k}\cdot S^{u,v}\left(t_{n},r_{k}\right)$$

where $t_{p^{\prime },q^{\prime },u,v}$ is the time delay result from the distance between the coded-aperture array element $\left(p^{\prime },q^{\prime }\right)$ and the antenna-array element (u,v). $S^{u,v}\left(t_{n},r_{k}\right)$ is the reference signal of the *k*-th grid cell at time $t_{n}$ for antenna-array element (u,v), which can be expressed as,

(9) $$S^{u,v}\left(t_{n},r_{k}\right)=\sum_{p^{\prime }=1}^{P}\sum_{q^{\prime }=1}^{Q}\sum_{p^{\prime }=1}^{P}\sum_{q^{\prime }=1}^{Q}A\cdot \exp\left\{j\cdot \left[\varphi_{p,q}^{\mathrm{purpose}}+\varphi_{p^{\prime },q^{\prime }}^{\mathrm{random}}+2\pi \left(f_{c}+f_{t_{n}}\right)\left(t_{n}-t_{Tx,p,q}-t_{p,q,k}-t_{k,p^{\prime },q^{\prime }}-t_{p^{\prime },q^{\prime },u,v}\right)\right]\right\}$$

Upon sampling the echo and obtaining N samples, the receiving signal for antenna-array element (u,v) can be rewritten as,

(10) $$Sr_{u,v}={\left[Sr^{u,v}\left(t_{1}\right),Sr^{u,v}\left(t_{2}\right),\ldots ,Sr^{u,v}\left(t_{N}\right)\right]}^{T}$$

Similarly, the reference-signal matrix corresponding to the antenna-array element (u,v) reads,

(11) $$S_{u,v}=\left[\begin{array}{cccc} S^{u,v}\left(t_{1},r_{1}\right) & S^{u,v}\left(t_{1},r_{2}\right) & \ldots & S^{u,v}\left(t_{1},r_{K}\right)\\ S^{u,v}\left(t_{2},r_{1}\right) & S^{u,v}\left(t_{2},r_{2}\right) & \ldots & S^{u,v}\left(t_{2},r_{K}\right)\\ \ldots & \ldots & \ldots & \ldots \\ S^{u,v}\left(t_{N},r_{1}\right) & S^{u,v}\left(t_{N},r_{2}\right) & \ldots & S^{u,v}\left(t_{N},r_{K}\right) \end{array}\right]$$

**Step 5. Signal Processing**

Next, we can combine all the reflected -signal vectors and reference-signal matrices together to generate the final reflected -signal vector and reference-signal matrix, which can be deduced as,

(12) $$Sr_{\mathrm{SIMO}}={\left[Sr_{1,1}{}^{T}\;\cdots \;Sr_{1,V}{}^{T}\;\cdots \;Sr_{u,v}{}^{T}\;\cdots \;Sr_{U,1}{}^{T}\;\cdots \;Sr_{U,V}{}^{T}\right]}^{T}$$

(13) $$S_{\mathrm{SIMO}}={\left[S_{1,1}{}^{T}\;\cdots \;S_{1,V}{}^{T}\;\cdots \;S_{u,v}{}^{T}\;\cdots \;S_{U,1}{}^{T}\;\cdots \;S_{U,V}{}^{T}\right]}^{T}$$

where ${\left[\cdot \right]}^{T}$ denotes matrix transposition. Eventually, the imaging model is deduced as,

(14) $$Sr_{\mathrm{SIMO}}=S_{\mathrm{SIMO}}\cdot \beta +w$$

As shown in Equations (1) and (14), to construct a reference-signal matrix with the same size, coding and sampling time of the common SISO TCAI architecture is U⋅V times more than that of the SIMO TCAI architecture. Thus, receiving array leads to a sharp reduction in coding and sampling time, which would vastly improve the imaging efficiency.

For the human-scale target in Figure 2, scattering coefficients β of each 3D imaging cell are reconstructed one by one to decompose the global computational complexity, and then are synthesized together to obtain the complete high-resolution image.

Compressive sensing (CS) is an efficient methodology to resolve sparse β from Equations (1) and (14). We adopt sparse Bayesian learning (SBL) [23] to reconstruct the 3D target in this paper.

## 3. Scanning and Imaging Method for 3D Target

As shown in Equation (4), by changing the focal length and the phase center coordinate of the designed digital lens, the beam direction can be digitally steered to illuminate the aimed 3D imaging cell. Thus, we describe the detailed scanning method in Section 3.1. Furthermore, for 3D high-resolution TCAI imaging, large-scale reference-signal matrix places heavy burden on computational complexity of the imaging. Even when the whole imaging area is divided into much smaller 3D imaging cell, size of the corresponding reference-signal matrix is still huge. For example, sizes of the grid cell and 3D imaging cell are 0.0025 m × 0.0025 m × 0.0025 m and 0.1 m × 0.1 m × 0.3 m, respectively, then the 3D imaging cell would include 192,000 grid cells. If the sampling ratio on time has no compression, size of the reference-signal matrix is over 192,000 × 192,000 and it would occupy 30 G computer memory, which requires much on computational ability. To improve the computational efficiency, we advance a multi-resolution imaging method in Section 3.2.

### 3.1. Scanning Method

As shown in Equation (4), the PPMF can be decomposed into PPMF for *x*-axis and *y*-axis direction, which is named after *x*-PPMF and *y*-PPMF, respectively. For coded-aperture array element (p,q), *x*-PPMF and *y*-PPMF read,

(15) $$\varphi_{x,p,q}^{\mathrm{purpose}}=e^{-i\frac{k}{2f_{\mathrm{focus}}}\left[{\left(x_{p,q}-x_{0}\right)}^{2}\right]}$$

(16) $$\varphi_{y,p,q}^{\mathrm{purpose}}=e^{-i\frac{k}{2f_{\mathrm{focus}}}\left[{\left(y_{p,q}-y_{0}\right)}^{2}\right]}$$

where $\varphi_{x,p,q}^{\mathrm{purpose}}$ and $\varphi_{y,p,q}^{\mathrm{purpose}}$ share the same focal length $f_{\mathrm{focus}}$ and wave number *k*.

The imaging area is divided into a M×N array of 3D imaging cells. Herein, the imaging cell (m,n) is used to denote the imaging cell of the *m*-th row, and *n*-th column. Take the imaging cell (m,n) as the aimed irradiation area, the scanning method can be simplified into three main steps, including preliminary solutions on *x*-PPMF and *y*-PPMF, modified solutions on *x*-PPMF and *y*-PPMF, final solution on PPMF.

**Step 1. Preliminary Solutions on *x*-PPMF and *y*-PPMF**

**Figure 4:**
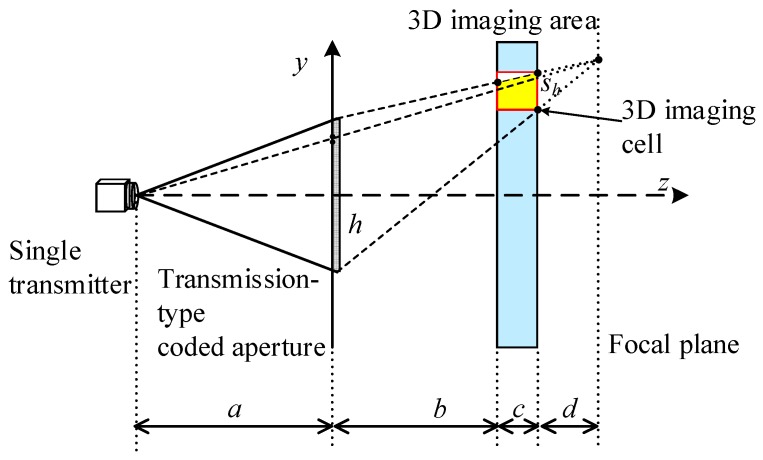
Schematic diagram of preliminary solution on the purposive phase modulation factor for *y*-axis direction, which can be abbreviated as *y*-PPMF.

The purpose of preliminary solutions is to direct the terahertz beam to cover the right face of the aimed imaging cell, which is shown as a red box in Figure 4. The solution of *y*-PPMF have no relationship with the *x*-direction parameters, so we project the 3D scene onto the *yoz* plane. Figure 4 illustrates the schematic diagram of preliminary solution on *y*-PPMF. The transmitter, the centers of coded aperture and the imaging plane are all on the *z* axis. *a* and *b* are the distances between the coded aperture and the transmitter, and the left face of the 3D imaging area, separately. *c* and $s_{h}$ are the thickness and height of the 3D imaging cell, respectively. *h* is the height of the coded aperture. For a TCAI system, *a*, *b*, *c*, $s_{h}$ and *h* are set to be known.

*d* is the distance between the focal plane and the right face of the imaging area, and it can be deduced as,

(17) $$d=\frac{s_{h}\cdot \left(b+c\right)}{h-s_{h}}$$

$f_{\min}^{y}$, the minimum focal length required for the *y* direction, can be expressed as,

(18) $$f_{\min}^{y}=\frac{a\cdot \left(b+c+d\right)}{a+b+c+d}$$

Preliminary *y* coordinate of the phase center reads,

(19) $$y_{0}^{m,n}=\frac{N+1-2n}{2}\cdot \Delta y_{0}$$

where $\Delta y_{0}$ is the *y*-coordinate difference of the phase centers between adjacent imaging cells, namely $\Delta y_{0}=\left|y_{0}^{m,n+1}-y_{0}^{m,n}\right|$, and it can be written as,

(20) $$\Delta y_{0}=\frac{a\cdot s_{h}\cdot \left(b+c+d\right)}{\left(a+b+c+d\right)\cdot \left(b+c\right)}$$

where $\Delta y_{0}$ is irrelevant to *m* and *n*.

Apparently, parameters of preliminary solution on *x*-PPMF, such as $f_{\min}^{x}$ and $x_{0}^{m,n}$, can also be worked out when we replace *h* and $s_{h}$ with *w* and $s_{w}$. *w* and $s_{w}$ are the widths of the coded aperture and the 3D imaging cell, respectively. In Equation (18), $f_{\min}^{y}$ contains neither *h* nor $s_{h}$. Thus, $f_{\min}^{x}$ is the same value as $f_{\min}^{y}$.

**Step 2. Modified Solutions on *x*-PPMF and *y*-PPMF**

**Figure 5:**
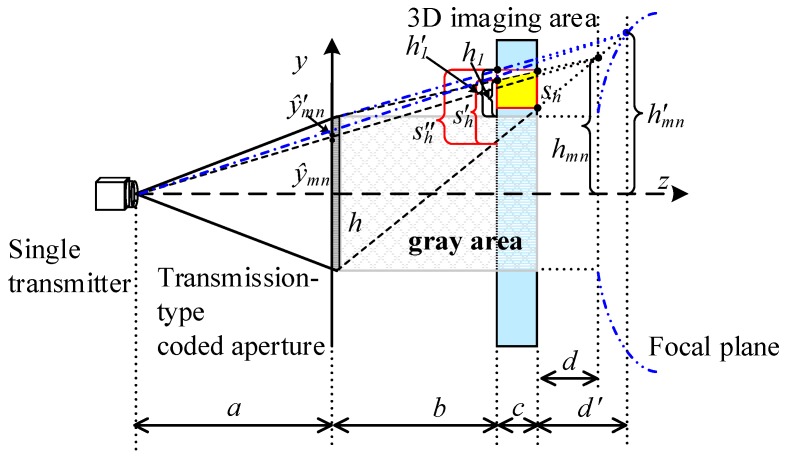
Schematic diagram of modified solution on *y*-PPMF.

Modified solutions on *x*-PPMF and *y*-PPMF aim to direct the terahertz beam to cover the entire imaging cell. Figure 5 depicts the schematic diagram of modified solution on *y*-PPMF. As shown in Figure 5, we define a rectangular gray area, the height of which is the same as that of the coded aperture. Moreover, the left and right sides of the gray area coincide with the coded aperture and the right face of the 3D imaging areas, respectively. As we know, the preliminary *x*-PPMF and *y*-PPMF can cover the right face of the imaging cell. If the imaging cell is in the gray area, it can be entirely illuminated with the preliminary solutions. Therefore, the preliminary solutions have no need for further modification while the gray area includes the aimed imaging cell.

If the gray area cannot include the aimed imaging cell, the preliminary solutions do not work for the left face of the imaging cell, which is shown in Figure 5. To achieve entire illumination, covering size on the left face needs to change from $s_{h}^{\prime }$ to $s_{h}^{\prime \prime }$. $s_{h}^{\prime }$ can be expressed as,

(21) $$s_{h}^{\prime }=\frac{\left(c+d\right)\cdot s_{h}}{d}$$

The difference between $s_{h}^{\prime }$ and $s_{h}^{\prime \prime }$ can be written as,

(22) $$\Delta s_{h}=\frac{c\cdot \left(\left|h_{m,n}\right|-h/2\right)}{b+c+d}$$

where $h_{m,n}$ is the *y* coordinate for the focus of the terahertz beam before modification. According to the relationship of similar triangles, $h_{m,n}$ can be deduced as,

(23) $$h_{m,n}=\frac{y_{0}^{m,n}\cdot \left(a+b+c+d\right)}{a}$$

In combination of Equations (21)–(23), we can get the value of $s_{h}^{\prime \prime }=s_{h}^{\prime }+\Delta s_{h}$.

Based on imaging formula of the digital lens, the minimum focal length required for the *y* direction is modified as,

(24) $${\tilde{f}}_{\min}^{y}=\frac{a\cdot \left(b+c+d^{\prime }\right)}{a+b+c+d^{\prime }}$$

where $d^{\prime }$ is the distance between the modified beam focus and the right face of the aimed imaging cell, and it can be written as,

(25) $$d^{\prime }=\frac{s_{h}^{\prime \prime }\cdot b+s_{h}^{\prime \prime }\cdot c-h\cdot c}{h-s_{h}^{\prime \prime }}$$

Moreover, modified *y* coordinate of the phase center reads,

(26) $${\tilde{y}}_{0}^{m,n}=\frac{a\cdot h_{m,n}^{\prime }}{a+b+c+d^{\prime }}$$

where $h_{m,n}^{\prime }$ is the *y* coordinate of the beam focus after modification, and it can be deduced as,

(27) hm,n′={h1′⋅(b+c+d′)b+h2 y^n>0−h1′⋅(b+c+d′)b−h2 y^n<0

As shown in Figure 5, the top edge of the beam intersects with the left side of the 3D imaging area. $h_{1}$ and $h_{1}^{\prime }$ are the distance between the gray area and the intersection points before and after modification. $h_{1}$ is expressed as,

(28) $$h_{1}=\frac{b\cdot \left(\left|v_{m,n}\right|-h/2\right)}{b+c+d}$$

while $h_{1}^{\prime }$ can be solved by $h_{1}^{\prime }=h_{1}+\Delta s_{h}$.

Similarly, ${\tilde{f}}_{\min}^{x}$ and ${\tilde{x}}_{0}^{m,n}$ can also be obtained by substituting *y*-direction parameters with corresponding *x*-direction parameters.

**Step 3. Final solution on PPMF**

As digital lens can only have one focal length, and the longer focal length helps the terahertz beam to cover larger area. So, the final focal length of the digital length can be chosen as,

(29) $$f_{\mathrm{focal}}=\max\left({\tilde{f}}_{\min}^{x},{\tilde{f}}_{\min}^{y}\right)$$

Moreover, phase center coordinate $\left(x_{0},y_{0}\right)$ is assigned as $\left(x_{0}^{m,n},y_{0}^{m,n}\right)$. Finally, the coded aperture can realize precise scanning on the aimed 3D imaging cell according to Equation (4).

### 3.2. Imaging Method

**Figure 6:**
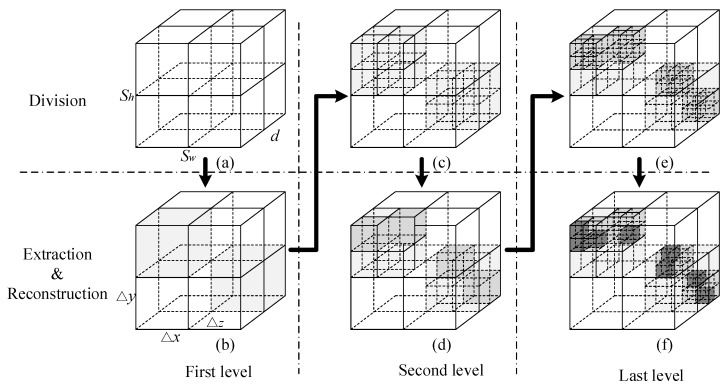
Basic process of three-level imaging.

Our multi-resolution imaging method aims to reduce the computational burden on large-scale reference-signal matrix for a 3D imaging cell. The total imaging process contains several resolution levels. Each level corresponds to one resolution. Moreover, the imaging resolution becomes higher and higher from the first to the last level. Besides, SBL algorithm is introduced to perform the reconstruction and extraction process. To be clear and simple, Figure 6 introduces the imaging method with three resolution levels. However, our imaging method includes but is not limited to three levels. The three columns, from the left to the right, illustrate the imaging process at the first, second, and last level, separately. Moreover, the first row shows the dividing process while the second row describe the extraction and reconstruction process at different resolution levels.

Figure 6a,b present the basic process of the first-level division, extraction and reconstruction. The whole cube shows a 3D imaging cell, the size of which is $s_{w}\times s_{h}\times d$ corresponding to its width, height, and thickness. In Figure 6a, the 3D imaging cell is divided according to Δx×Δy×Δz, which is the size of the first-level grid cell. On the one hand, $s_{w}$, $s_{h}$, and d have to be dividable by Δx, Δy, and Δz, respectively. On the other hand, for each imaging cell, the number of first-level grid cells needs to be set reasonable for constructing the first-level reference-signal matrix $S_{1}$, which can be deduced from Equations (9), (11), and (13). Actually, each 3D imaging cell usually contains hundreds of grid cells. However, to show the dividing process clearly, Figure 6a simply divides the 3D imaging cell into eight first-level gird cells. In Figure 6b, referring to Equation (1), we can reconstruct the first-level scattering coefficients $\beta_{1}$ according to $Sr=S_{1}\cdot \beta_{1}+w$.

To reduce the computational burden, only the grid cells containing objects in the first-level will be further extracted and divided for the second and last levels. Herein, we define a threshold $\beta_{\mathrm{limit}}$ to extract the grid cells with scattering information, where $\beta_{\mathrm{limit}}$ is about one percent of the maximal vector-element of $\beta_{1}$. As shown in Figure 6b, when the coefficient element $\beta_{1}^{i}>\beta_{\mathrm{limit}},\;i=1,2,\cdots ,K$, it is extracted and indexed as gray while other useless ones are eliminated.

The imaging process of the second and last levels are similar to that of the first level. We divide the extracted cells at the second and last levels considering the size of 0.5Δx×0.5Δy×0.5Δz and 0.25Δx×0.25Δy×0.25Δz, respectively. In Figure 6f, we can construct the final reference-signal matrix $S_{3}$, which has been greatly simplified after previous reconstructions and extractions. Eventually, high-resolution imaging can be realized by solution of $Sr=S_{3}\cdot \beta_{3}+w$. The scattering coefficients $\beta_{3}$ are painted with the darkest gray, which is shown in Figure 6f. For the same resolution, the traditional coded-aperture imaging would directly divide the imaging cell into 0.25Δx×0.25Δy×0.25Δz grid cells, which lead to large-scale reference-signal matrix.

## 4. Numerical Simulations

In this section, firstly, we would compare and analyze the resolving ability of TCAI based on SIMO and common SISO technique by space independence function [24]. Next, to analyze the performance of digital beam scanning for 3D TCAI imaging, radiation field distributions on both the left and right faces of the imaging area are presented for different aimed 3D imaging cells. Then, we convolve the radiation patterns with a meshed 3D human target to deduce the simulated TCAI reflected signals, based on which, we can reconstruct the target with our multi-resolution imaging method. Moreover, we have compared the imaging results using our SIMO TCAI technology and common SISO TCAI approach, which would consume much more time.

The primary parameters used in the simulations are given in Table 1. The largest size of the *xoy* imaging plane is 2 m × 2 m. To reduce the scanning time, we downsize the imaging plane to *x* × *y* = 0.8 m × 1.8 m according to the size of an ordinary human. As shown in Table 1, the distances between the coded aperture and the transceiver, and the imaging area are 0.25 m and 0.6 m, respectively. So, the imaging distance can be calculated as 0.85 m.

### 4.1. Resolving Ability Analysis

**Figure 7:**
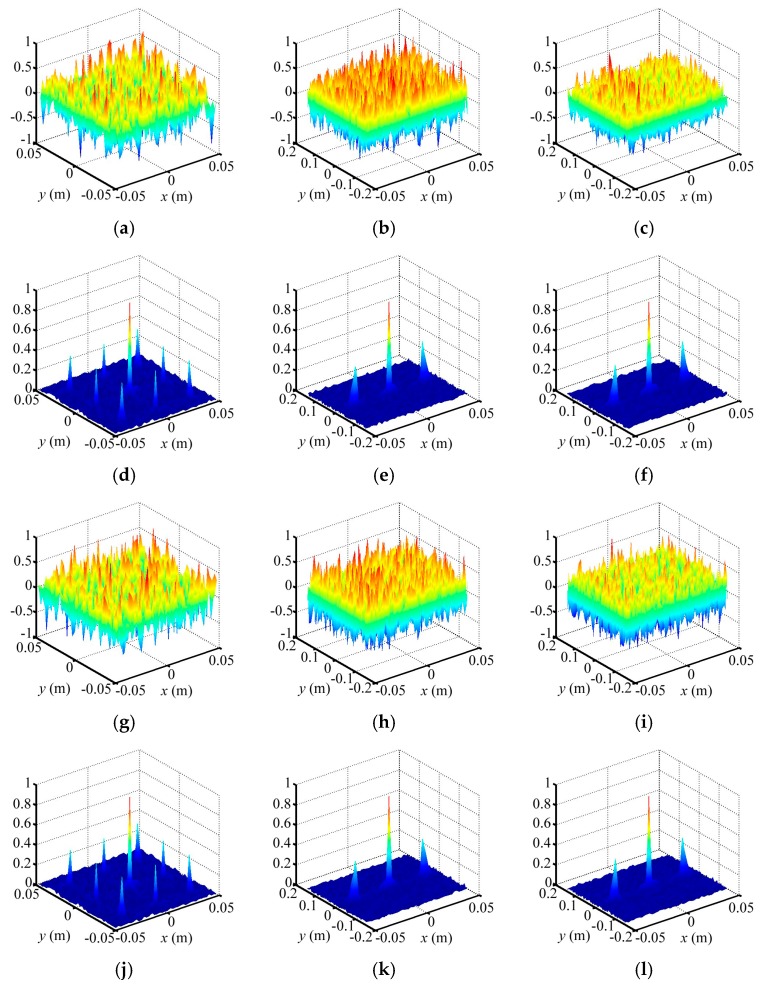
(**a**–**c**) Radiation field distributions for SIMO TCAI corresponding to *xoy*, *xoz*, and *yoz*, respectively; (**d**–**f**) Space independence functions for SIMO TCAI corresponding to *xoy*, *xoz*, and *yoz*, respectively; (**g**–**i**) Radiation field distributions for SISO TCAI corresponding to *xoy*, *xoz*, and *yoz*, respectively; (**j**–**l**) Space independence functions for SISO TCAI corresponding to *xoy*, *xoz*, and *yoz*, respectively.

In this section, we use space independence function $\gamma_{\mathrm{space}}$ to describe the TCAI resolving, which is inverse proportional to $\gamma_{\mathrm{space}}$. Herein, we present a 0.5 m × 0.5 m SIMO array antenna with 10 × 10 receiving detectors. In contrast with the common SISO TCAI system, this architecture aims to save time with array of receiving detectors. Besides, we hope it can maintain its resolving ability as the SISO one. Thus, we adopt space independence functions of the reference-signal matrix to compare their resolving ability. In Figure 7, row numbers of the reference-signal matrices for both SIMO and SISO TCAI is 3000. However, the SIMO TCAI only needs 30 coding and sampling times while the SIMO TCAI requires 3000 times. Figure 7a–c,g–i depict the radiation field distributions for SIMO and SISO architecture corresponding to different planes of the aimed 3D imaging cell, respectively. Figure 7d–f,j,k present the space independence functions for SIMO and SISO architecture related to different planes of the aimed 3D imaging cell, separately. Radiation field distributions and space independence functions corresponding to *xoy*, *xoz*, and *yoz* planes of the aimed 3D imaging plane can describe imaging ability considering the azimuth, range, and pitching dimensions. As shown in Figure 7a–c,g–i, random fluctuations for SIMO and SISO in the three dimensions are almost the same as each other. Besides, space independence functions in Figure 7d–f,j–l demonstrate their close imaging ability further. Thus, we suppose that SIMO TCAI technology performs as well as SISO one with much fewer coding and sampling times.

### 4.2. Scanning Ability Analysis

**Figure 8:**
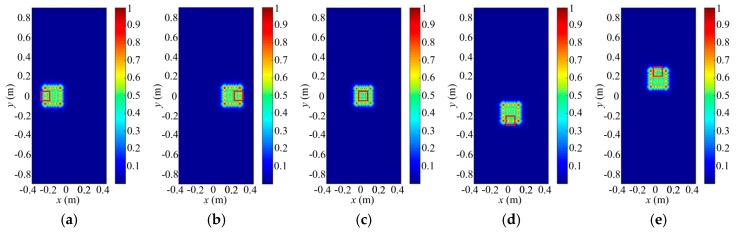
Distributions of incident fields on the left face of the imaging area for different 3D imaging cells with different PPMFs (*f*_focal_@(*x*_0_, *y*_0_)): (**a**) 0.2069@(−0.0603, 0); (**b**) 0.2069@(0.0603, 0); (**c**) 0.2046@(0, 0); (**d**) 0.2069@(0, −0.0603); and (**e**) 0.2069@(0, 0.0603).

**Figure 9:**
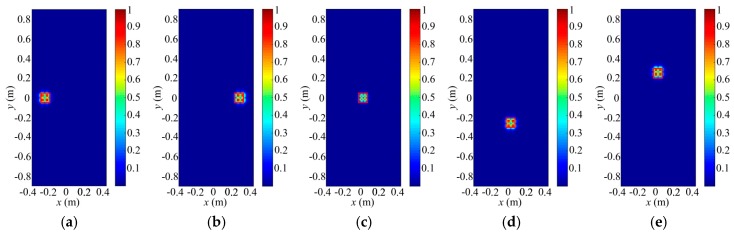
Distributions of incident fields on the right face of the imaging area for different 3D imaging cells with different PPMFs (*f*_focal_@(*x*_0_, *y*_0_)): (**a**) 0.2069@(−0.0603, 0); (**b**) 0.2069@(0.0603, 0); (**c**) 0.2046@(0, 0); (**d**) 0.2069@(0, −0.0603); and (**e**) 0.2069@(0, 0.0603).

Next, we would analyze and verify the scanning ability for the aimed 3D imaging cell by using proper PPMF, which can be designed and modified by the scanning method in Section 3.1. Figure 8 and Figure 9 illustrate the distributions of the incident field on the left and right faces of the imaging area for different 3D imaging cells with different PPMFs. As shown in Figure 8c and Figure 9c, the aimed 3D imaging cell is located in the center of the imaging area, which is entirely included in the gray area. Thus, the PPMF can be obtained without modification. Its focal length and phase center of the digital lens is 0.2046 and (0, 0), respectively. Apparently, both the left and right faces of the imaging cell are illuminated by the terahertz wave. In contrast, aimed 3D imaging cells in Figure 8a,b,d,e or Figure 9a,b,d,e are not contained in the gray area. Besides the preliminary solutions, they need further modification. After modified solutions, all their focal lengths change from 0.2046 to 0.2069, and their phase-center coordinates transform from (−0.0568, 0), (0.0568, 0), (0, −0.0568) and (0, 0.0568) to (−0.0603, 0), (0.0603, 0), (0, −0.0603), (0, 0.0603), respectively. Before modification solution, their left faces cannot be wholly covered by the beams. By further modification, both their left and right faces are covered by the redirected terahertz waves. According to the principle that light travels in straight line, the whole imaging cell can be illuminated with the designed PPMF. Eventually, it is demonstrated that our scanning method really works.

### 4.3. Working-Distance Analysis

**Figure 10:**
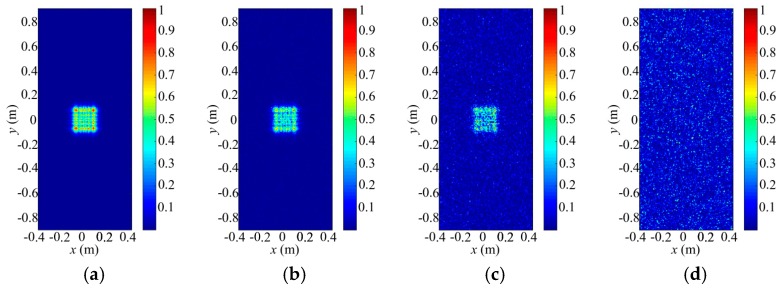
Distributions of the random-modulation incident fields on the left face of the imaging area with the same PPMF and different RPMF phase ranges: (**a**) [−0.25π, 0.25π]; (**b**) [−0.5π, 0.5π]; (**c**) [−0.75π, 0.75π]; and (**d**) [−π, π].

As shown in Figure 8c, the beam energy focuses on the center region of the left face for the imaging area. To analyze the working-distance superiority for our imaging architecture, we add to the transmitting signal with different RPMF phase ranges, which contain [−0.25π, 0.25π], [−0.5π, 0.5π], [−0.75π, 0.75π], and [−π, π]. Then, the distributions of the random-modulation incident fields are presented in Figure 10. Obviously, RPMFs adding in the transmitting process result in energy dispersion on the imaging plane. Moreover, the energy-dispersion degrees increase with the expansion of the RPMF phase ranges. The beam direction is completely destroyed until the RPMF phase range gets [−π, π]. As for our imaging method, The RPMFs in the proposed imaging architecture only work in the receiving process without damaging the transmitting beam-energy distribution, which is shown in Figure 8c. Therefore, our imaging architecture can achieve better working-distance performance than some common ones, which form the spatial-temporal independent signals in the transmitting process.

### 4.4. Imaging Results

**Figure 11:**
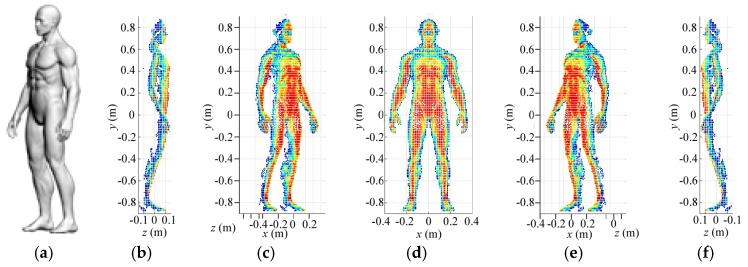
(**a**) The 3D human target, and reconstruction results at low-resolution level in different perspectives: (**b**) the left side; (**c**) the left front side; (**d**) the front; (**e**) the right front side; and (**f**) the right side.

In this section, we adopt a 3D human target, which is shown in Figure 11a. According to the THz wavelength at 340 GHz, the human target is meshed into thousands of triangle facets by computer simulation technology (CST) software. Different facets are given scattering coefficients corresponding to angles of their normal vectors and the irradiation directions. The reflected signals for radar coded-aperture imaging are simulated by convolution of the scattering coefficients and the coded-aperture radiation patterns. Furthermore, the SBL algorithm is introduced to process the reflected-signal vector and pre-constructed reference-signal matrix. The 3D imaging area is subdivided into 144 3D imaging cells. To reduce the computational complexity, we would reconstruct the scattering information in each imaging cell gradually, and then synthesize each part of the human target together. The experiments are performed on a computer with Intel Core CPU i5-6200U at 2.3 GHz and 8 GB of memory.

Firstly, Figure 11 illustrates the reconstruction results for low-resolution level in different perspectives. Size of the 3D imaging cell and grid cell at the first level is 0.1 m × 0.1 m × 0.3 m and 0.01 m × 0.01 m × 0.01 m, respectively. Thus, the imaging cell contains 3000 low-resolution cells, and size of the constructed reference-signal matrix is 3000 × 3000, which occupy little computer memory. As shown in Figure 11, with a proper threshold, the useful low-resolution grid cells are extracted and painted in different colors based on the reconstruction results. Moreover, the other gird cells are eliminated to save computer memory for further imaging.

**Figure 12:**
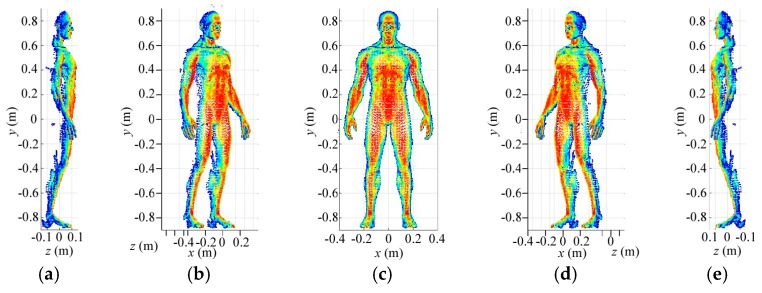
Reconstruction results at medium-resolution level in different perspectives: (**a**) the left side; (**b**) the left front side; (**c**) the front; (**d**) the right front side; and (**e**) the right side.

Secondly, Figure 12 presents the reconstruction results for medium-resolution level in different perspectives. Size of the medium-resolution grid cell is 0.005 m × 0.005 m × 0.005 m, according to which, the selected grid cells in low-resolution reconstruction is divided further to build new reference-signal matrix. Fortunately, number of the medium-resolution grid cells still keeps proper for the size of the matrix. Similarly, as shown in Figure 12, the useful grid cells are picked out and the useless ones are discarded. Then, the imaging results in Figure 12 become much clearer than that in Figure 11.

**Figure 13:**
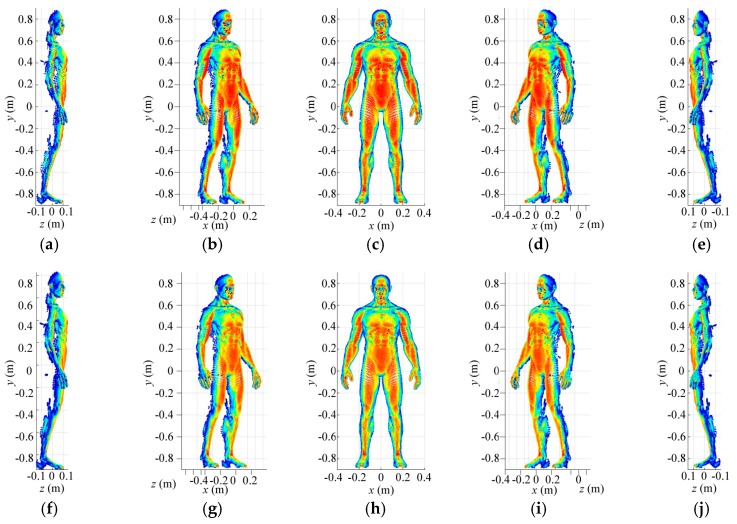
(**a**–**e**) Imaging results at high-resolution level for SIMO architecture in different perspectives; (**f**–**j**) Imaging results at high-resolution level for SISO architecture in different perspectives.

Eventually, Figure 13a–e show the high-resolution imaging results in different perspectives. The imaging resolutions corresponding to azimuth, pitching and range are 0.0025 m × 0.0025 m × 0.0025 m, which is the size of the high-resolution grid cell. The high-resolution reference-signal matrix is built by high-resolution grid cells subdivided from the extracted medium-resolution grid cells. For high-resolution imaging, number of rows for the reference-signal matrix is still 3000 while its column number is determined by the amount of the final grid cells. Although the constructed reference-signal matrices vary with number of the grid cells in different resolution levels, the coding and sampling times maintain 30. Therefore, the reflected-signal vector for a 3D imaging cell only needs to be obtained once for multi-resolution imaging, which consumes no more time for high-resolution 3D imaging. As shown in Figure 13a–e, except for some blurred points, the imaging results present much more details than that in Figure 11 and Figure 12. Moreover, Figure 13f–j display imaging results for SISO TCAI system under the same parameters with 3000 coding and sampling times. Both architectures can reconstruct the 3D human target well with our scanning and imaging method, and their imaging results are similar to each other. However, the SISO would take 100 times the sampling time than our architecture. As each 3D imaging cell includes 19,200 high-resolution grid cells, common method will operate on all 19,200 grid cells without discrimination. As for our method, each imaging cell is pre-divided with 0.01 m × 0.01 m × 0.01 m low-resolution grid cell at first, so the grid cells needed operation are only 3000. When it comes to higher resolution level, our method only operates with the useful high-resolution grid cells, so the 3D imaging cell without target information needs no further processes, which saves much more time. Under the computer environment mentioned above, reconstruction time for each level is about 2 min. With three resolution levels, the total imaging time is around 6 min. For the common imaging method under the same architecture, it cannot perform on the same computer environment. Moreover, the computation can be parallelized using high-performance graphical processing units (GPUs), so that the imaging time with our method can be further reduced sharply. Even with GPUs and higher computer performance, similar 3D human imaging [8] still needs several minutes for 0.0054 m × 0.0054 m × 0.0167 m resolution, which is much lower than that in our TCAI system. In conclusion, the SIMO TCAI architecture achieves fast imaging with high resolution.

## 5. Conclusions

This paper proposed a 3D TCAI architecture based on SIMO technology to achieve the performance of high-resolution, forward-looking, and fast imaging. According to the basic principle of terahertz propagation, we built the mathematical imaging model for the proposed SIMO TCAI architecture. Besides, space independence function was adopted to estimate the resolving ability of the TCAI architecture qualitatively. We advanced a 3D-target scanning method by designing and modulating the PPMFs for beam direction control. RPMFs only work in the receiving process without disturbing the transmitting signal, which is helpful for improving the working distance. Furthermore, our imaging method transfers computational burden on large-scale reference-signal matrix to several resolution levels. Finally, numerical experimental results have demonstrated that our architecture could achieve: (1) fast imaging with much less coding and sampling times; (2) rapid digital scanning by designed PPMFs; (3) longer working distance owing to no random phase modulations in the transmitting process; (4) high-resolution and memory-saving imaging based on multi-resolution method. In conclusion, our proposed architecture could be a promising 3D radar imager, which holds potential applications in areas such as terminal guidance, security check, etc.

## Figures and Tables

**Table 1:** Primary parameters used in the simulations.

Parameter	Value
Center frequency (*f_c_*)	340 GHz
Bandwidth (*B*)	20 GHz
Sampling frequency (*f_s_*)	10 MHz
Distance between coded aperture and transceiver (*a*)	0.25 m
Distance between coded aperture and left face of the imaging area (*b*)	0.6 m
Coding and sampling times	30
Number of the receiving detectors	10 × 10
Size of the SIMO array antenna	0.5 m × 0.5 m
Size of the 3D imaging cell (*s_w_* × *s_h_* × *d*)	0.1 m × 0.1 m × 0.3 m
Size of the first-level grid cell	0.01 m × 0.01 m × 0.01 m
Size of the second-level grid cell	0.005 m × 0.005 m × 0.005 m
Size of the last-level grid cell	0.0025 m × 0.0025 m × 0.0025 m
Size of the transmission-type coded aperture (*h* × *v*)	0.5 m × 0.5 m
Range of random phase ($p_{l}$, $p_{h}$)	(−0.75π, 0.75π)
